# Mitochondrial cytochrome c oxidase subunit I gene analysis of the yellowfin snapper, *Lutjanus xanthopinnis* in the Indo-Pacific region and a note on *Lutjanus lutjanus* population structure

**DOI:** 10.1016/j.heliyon.2023.e19348

**Published:** 2023-08-22

**Authors:** Takaomi Arai, Hussein Taha, Najihah Alidon, Juhaidah Jumat, Syakirah Azmey, Nur Dhabitah Zan, Tun Nurul Aimi Mat Jaafar, Ahasan Habib

**Affiliations:** a Environmental and Life Sciences Programme, Faculty of Science, Universiti Brunei Darussalam, Jalan Tungku Link, Gadong, BE1410, Brunei Darussalam; b Faculty of Fisheries and Food Science, Universiti Malaysia Terengganu, Kuala Nerus, Terengganu, 21030, Malaysia

**Keywords:** Biogeography, Commercial fishes, Diversity, DNA barcoding, Lutjanidae, Migration

## Abstract

The yellowfin snapper, *Lutjanus xanthopinnis*, was recorded as a newly described species in the Indo-Pacific region in 2015. However, the knowledge of its biology, biogeography and ecology is scarcely understood, and, hence, its current conservation status is categorized as Data Deficient. The mitochondrial cytochrome *c* oxidase subunit I (COI) gene was examined to confirm species identification. We also examined the COI gene haplotypes of *L. xanthopinnis* in Brunei Darussalam and Malaysia together with other waters, i.e., Bangladesh, Indonesia, Japan, Singapore, Sri Lanka and Taiwan. Our molecular analyses found that Brunei Darussalam and eastern Peninsular Malaysia samples were genetically similar. However, the former showed higher genetic diversity than the latter. The samples from these two sites also showed signatures of population expansion. Furthermore, identical haplotypes could be found in different locations, suggesting the absence of spatial genetic structure. On the other hand, *Lutjanus lutjanus* showed a population structure associated with geographical locations, i.e., western Pacific Ocean, Indian Ocean and Maluku in Indonesia.

## 1 Introduction

Snappers, family Lutjanidae, are very diverse fisheries resources with 17 genera and 113 species, and are globally distributed in the Atlantic and Indo-Pacific subtropical and tropical waters [1]. Within the family, the genus *Lutjanus* is the most speciose with 73 species [1], and is considered as ecologically and economically important fishery resource within the distribution ranges. Snappers are widely found in coral reefs, rocky shore, near shore and offshore habitats, and are the most common predatory species in coral reef ecosystems [2]. In addition, they are slow growing, long-lived and considered significant food resources in Brunei Darussalam and Malaysia [3].

Several scientists have revised the taxonomy of *Lutjanus* based on their external morphological features and meristic counts. However, due to their morphological similarity and overlapping diagnostic characteristics, their taxonomy is still ambiguous and confusing, and the phylogenetic relationships of *Lutjanus* species remain obscure [[4], [5], [6]]. Accurate species identification is essential for further research on life history, stock assessment and stock management [7].

Indeed, the yellow-lined *Lutjanus* received recent attention from Ref. [4] and a new species, the yellowfin snapper *Lutjanus xanthopinnis*, was subsequently discovered. This is not surprising because *L. xanthopinnis* resembles *Lutjanus madras*; hence, it was previously mistaken as this species. In addition, *L. madras* also exhibits similar colour patterns with other members of the yellow-lined snapper complex including *Lutjanus lutjanus* and *Lutjanus vitta*. The discovery of *L. xanthopinnis* was made after the reexamination of the specimens collected in Indonesia, Japan, Sri Lanka and Taiwan, which was carried out using morphological characteristics, morphometric counts and DNA barcoding. The redescription of true *L. madras* also had to be made in the study [4]. In such a case, fundamental information regarding the biology, biogeography, ecology and conservation status of *L. xanthopinnis* is highly limited. Therefore, the current conservation status of *L. xanthopinnis* in the IUCN Red List is categorized as Data Deficient [8]. Furthermore, the population genetic structure of *L. xanthopinnis* is still undetermined, hampering pertinent conservation measures for this species. This task is crucial and requires immediate attention since lutjanids represent some of the most beneficial fisheries resources, contributing to their substantial exploitation. Furthermore, snappers also constitute an essential food resource for communities that rely on artisanal fisheries [9].

The bigeye snapper *L. lutjanus* is distributed in the tropical waters of the Indo-West Pacific region, and its distribution range overlaps with that of *L. xanthopinnis* [2,4]. Although *L*. *lutjanus* is a target species in fisheries and broadly consumed in this region, only a few research on its biology and ecology have been conducted [10]. In addition, although two divergent groups of *L. lutjanus* were found between the western and eastern Peninsular Malaysia (West Malaysia) through DNA barcoding [11], the population structure of *L. lutjanus* within the distribution range is not well examined.

The aim of the present study was to analyse the mitochondrial cytochrome *c* oxidase subunit I (COI) gene sequence of *L. xanthopinnis* in Brunei Darussalam and eastern Peninsular Malaysia. Mitochondrial DNA markers, particularly COI, have been proven to be a powerful tool for revealing phylogenetic patterns, species identification and genetic diversity of aquatic species [12,13]. Furthermore, the COI gene is commonly used for DNA barcoding, and is also useful for distinguishing cryptic species [11,14]. This study was also aimed to compare the COI sequences with the GenBank sequences of *L. xanthopinnis* from other sites (Bangladesh, Indonesia, Japan, western Peninsular Malaysia, Singapore, Sri Lanka and Taiwan), which would provide an insight into its population structure. We also similarly examined its closely related species, *L. lutjanus*.

## 2 Materials and methods

### 2.1 Fish samples

**Fig. 1:**
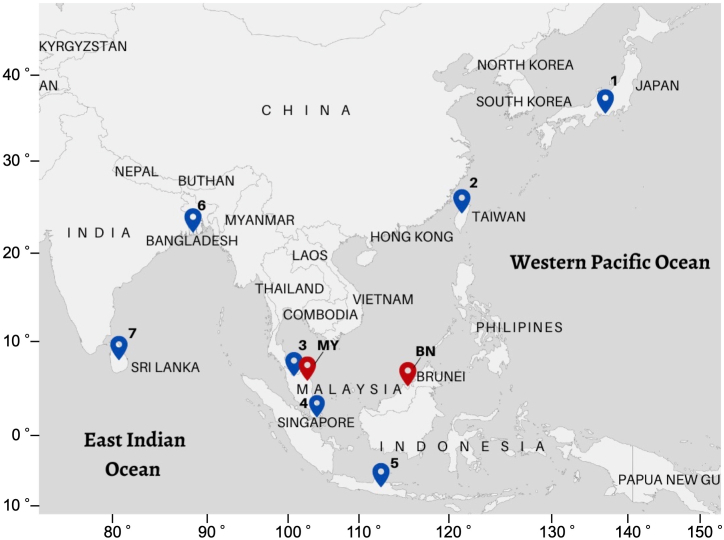
Map showing the collection sites of *Lutjanus xanthopinnis* in the Indo-west Pacific region. The red symbols indicate the sampling locations in Brunei Darussalam (BN) and Terengganu, eastern Peninsular Malaysia (MY). The blue symbols indicate the samples from the GenBank database: (1) Japan, (2) Taiwan, (3) western Peninsular Malaysia, (4) Singapore, (5) Indonesia, (6) Bangladesh, and (7) Sri Lanka. The base map was created using Map Chart at https://www.mapchart.net/world-pacific.html.

Thirty-three and 20 specimens were collected in the coastal waters of Brunei Darussalam in January 2021 and in the coastal waters of Terengganu, eastern Peninsular Malaysia, Malaysia in January 2021, respectivelly, and 15 specimens/sequences of *L. xanthopinnis* from seven countries, i.e., Bangladesh, Indonesia, Japan, Malaysia, Singapore, Sri Lanka, and Taiwan were obtained from the GenBank database (Fig. 1, Table 1). One ambiguous *L*. *xanthopinnis* sequence from the GenBank database was not included. The fish specimens were morphologically identified according to Iwatsuki et al. [4]*.* After measuring body weight and total length of each fish, all the fins, except the caudal fin, and muscle tissues were removed and preserved in absolute ethanol before further analysis. Our protocols followed the ethical guidelines for the use of animals of Universiti Brunei Darussalam (UBD) and were approved by the animal ethics committee at UBD.

**Table 1:** List of haplotypes for *L. xanthopinnis.*

Haplotype	No. of samples	Localities (sample ID or GenBank accession number)
H1	16	Brunei Darussalam (LX2, LX5, LX17, LX18, LV10, LV13, LV14), Sri Lanka (LC075756), Japan (LC071439), Bangladesh (MK027395), western Peninsular Malaysia: Perlis (MG002628), eastern Peninsular Malaysia: Terengganu (TGL26, TGL27, TGL31, TGL34, TGL54)
H2	24	Brunei Darussalam (LX1, LX3, LX4, LX6-LX8, LX10, LX12, LX13, LX15), Taiwan (LC071441, LC071442), Indonesia: Bali (JN311964), eastern Peninsular Malaysia: Terengganu (TGL24, TGL29, TGL32, TGL37, TGL40, TGL47- TGL49, TGL51-TGL53)
H3	1	Brunei Darussalam (LX9)
H4	5	Brunei Darussalam (LX11, LV7), Bangladesh (MG571550), eastern Peninsular Malaysia: Terengganu (TGL38, TGL50)
H5	1	Brunei Darussalam (LX14)
H6	1	Brunei Darussalam (LX16)
H7	1	Brunei Darussalam (LV6)
H8	2	Brunei Darussalam (LV15), Singapore (MZ314869)
H9	1	eastern Peninsular Malaysia: Terengganu (TGL33)
H10	1	eastern Peninsular Malaysia: Terengganu (TGL39)
H11	1	Bangladesh (MK064227)
H12	1	Bangladesh (MK340656)
H13	2	Bangladesh (MK340655, MK340657)
H14	1	Indonesia: Bali (JN311974)
H15	1	Sri Lanka (LC075755)

### 2.2 Mitochondrial DNA analysis

Genomic DNA was extracted using the QIAGEN DNeasy Blood & Tissue Kit according to the manufacturer's instructions. The partial mitochondrial COI gene was amplified by polymerase chain reaction (PCR) using the QIAGEN 2× *Taq* PCR Master Mix according to the manufacturer's instructions. The PCR primers were fish universal primers [15]. The PCR products were purified using the QIAGEN QIAquick Gel Extraction Kit. The purified COI fragments were sent to a sequencing service provider, where they were sequenced bi-directionally with the same primers.

MEGA X [16] was used to align, inspect and edit forward and reverse sequences. The resulting contig sequences were analysed and uploaded to the GenBank database. To identify the species via DNA barcoding, a BLAST [17] search in the GenBank database was used to match the contig sequences with the GenBank reference sequences. MEGA X was used to carry out multiple sequence alignment via ClustalW. The multiple alignment was trimmed at both ends to remove columns with missing data. Genetic distance was calculated using p distance model. A phylogenetic tree was constructed via the Maximum-Likelihood (ML) algorithm using the best-fit model (K2 + G model). A ML heuristic search starting with the initial NJ/BioNJ tree was conducted using the Nearest-Neighbor-Interchange method, and the ML tree was bootstrapped with 1000 replicates. DnaSP6 [18] was used for haplotype analysis and for constructing a mismatch distribution plot. Arlequin 3.5 [19] was used for the analysis of molecular variance (AMOVA), fixation index (F_ST_), sum of squared deviation (SSD), Harpending's raggedness index and neutrality tests. Network 10 (www.fluxus-engineering.com) was used to construct haplotype network using the median-joining method.

## 3 Results

This study collected and sequenced a total of 53 specimens form Brunei Darussalam and eastern Peninsular Malaysia. Initial morphological identification suggested that the specimens were mostly *L. xanthopinnis* (44 specimens) and a few *L. vitta* (9 specimens). With DNA barcoding, the BLAST results confirmed that 38 of 44 specimens were *L. xanthopinnis* with 99–100% identity. The other specimens were morphologically misidentified, in which 6 specimens were barcoded as *L. xanthopinnis* instead of *L. vitta*, and 9 specimens were barcoded as *L. lutjanus* instead of *L. xanthopinnis* or *L. vitta*, with 99–100% identity. Therefore, of 53 specimens, this study barcoded 44 specimens as *L. xanthopinnis* (GenBank accession no. OQ081715-OQ081758) and 9 specimens as *L. lutjanus* (OQ081759-OQ081767).

Based on the 573 bp of COI gene sequence, the samples from Brunei Darussalam showed a higher number of polymorphic sites (10 sites) than those from eastern Peninsular Malaysia (4 sites). Similarly, the haplotype diversity were 0.76 and 0.65, respectively, with the former having 8 haplotypes and the latter having 5 haplotypes. The within-group mean genetic distances (also known as nucleotide diversity, π) were 0.0025 and 0.0016, respectively. AMOVA resulted in a negative value for the within-group variation (−1.24%) and a value of 101.24% for the between-group variation. A pairwise comparison of the two collection sites showed that the samples were not significantly different from each other (fixation index, F_ST_ = −0.01, p > 0.05), suggesting that they were of one genetic population in the South China Sea.

**Fig. 2:**
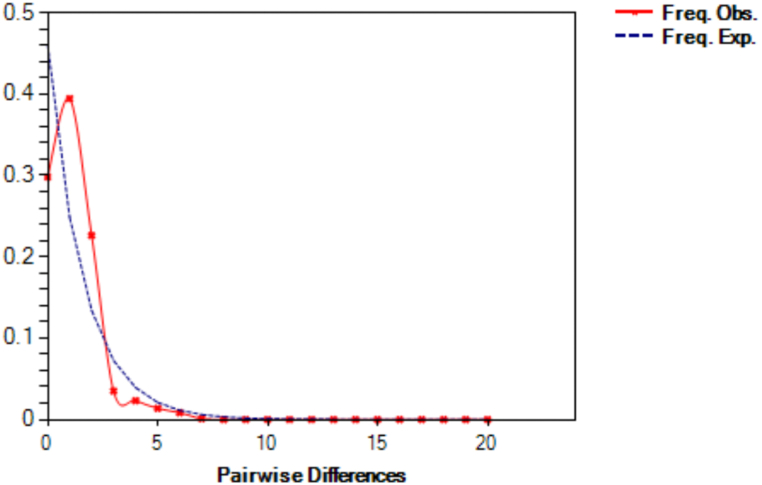
Mismatch distribution of *Lutjanus xanthopinnis* from Brunei Darussalam and eastern Peninsular Malaysia.

The 44 specimens of *L. xanthopinnis* were analysed as one genetic population. Historical demography was inferred based on the observed mismatch distribution, and the resulting mismatch distribution plot for this genetic population showed a unimodal shape (Fig. 2). The value for the sum of squared deviation (SSD) was not significant (0.0083; p > 0.05). The Harpending's raggedness index was also not significant (0.0744; p > 0.05). Neutrality tests showed negative and significant values for Tajima's D (−1.74; p < 0.05) and Fu's Fs (−4.84; p < 0.02), which support the notion that this population had undergone a population expansion.

**Fig. 3:**
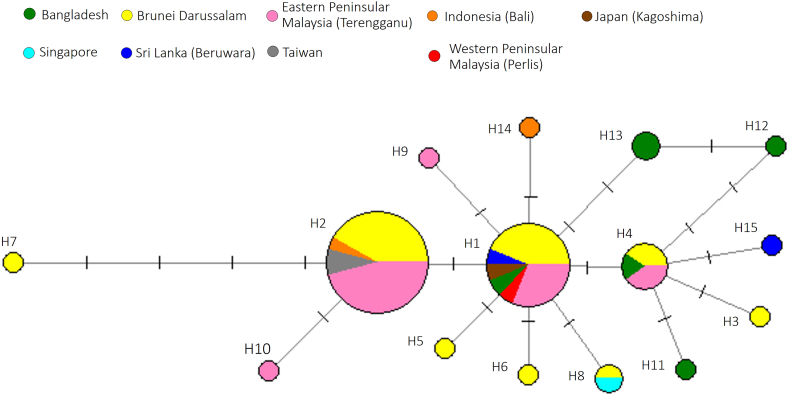
Haplotype network of *Lutjanus xanthopinnis* using the mitochondrial cytochrome *c* oxidase subunit I (COI) gene sequences. Different colours represent different geographical locations. Each dash, which appears on the line that connects two haplotypes, symbolises one mutational step. The circle size is proportional to the haplotype frequency.

**Fig. 4:**
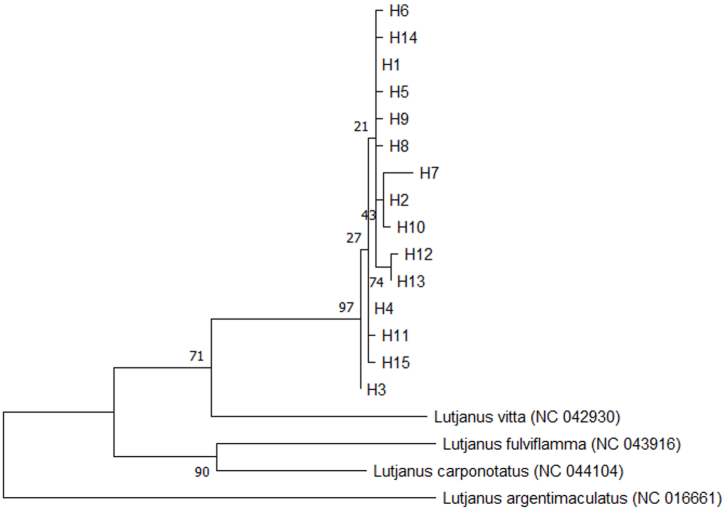
Maximum likelihood (ML) tree using the 15 haplotypes of *Lutjanus xanthopinnis*. For outgroups, *Lutjanus vitta, Lutjanus fulviflamma, Lutjanus carponotatus, and Lutjanus argentimaculatus* were used, with their GenBank accession numbers shown in parentheses. Bootstrap percentages are shown at the node.

Haplotype analysis of *L. xanthopinnis* found 15 haplotypes, each with a length of 511 bp (H1–H15 in Table 1 and Fig. 3). Two haplotypes, H1 and H2, were relatively dominant by having 16 and 24 samples, respectively, whereas other haplotypes only had 1 to 5 samples only. Interestingly, 4 haplotypes (H1, H2, H4 and H8) were shared by at least 2 geographically distant localities. For example, H1 was shared by Brunei, Bangladesh, Japan, eastern and western Peninsular Malaysia, and Sri Lanka. In addition, no distinct genetic grouping was observed from the ML tree of *L. xanthopinnis* using the 511 bp COI gene sequences (Fig. 4).

Based on the 574 bp of COI gene sequence, 5 polymorphic sites and 6 haplotypes were identified in *L. lutjanus* from Brunei Darussalam. The haplotype diversity and the nucleotide diversity (π) were 0.83 and 0.0019, respectively, which was almost similar to the nucleotide diversity of *L. xanthopinnis*.

**Fig. 5:**
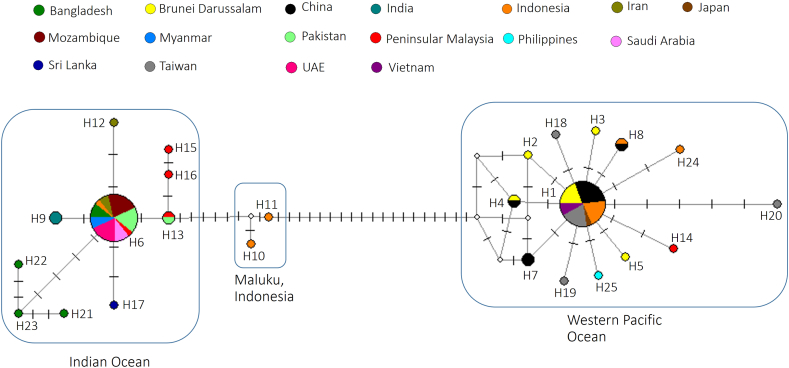
Haplotype network of *Lutjanus lutjanus* using the mitochondrial cytochrome *c* oxidase subunit I (COI) gene sequences. Different colours represent different geographical locations. Each dash, which appears on the line that connects two haplotypes, symbolises one mutational step. The circle size is proportional to the haplotype frequency. The small white circle represents hypothesized or missing haplotype.

**Fig. 6:**
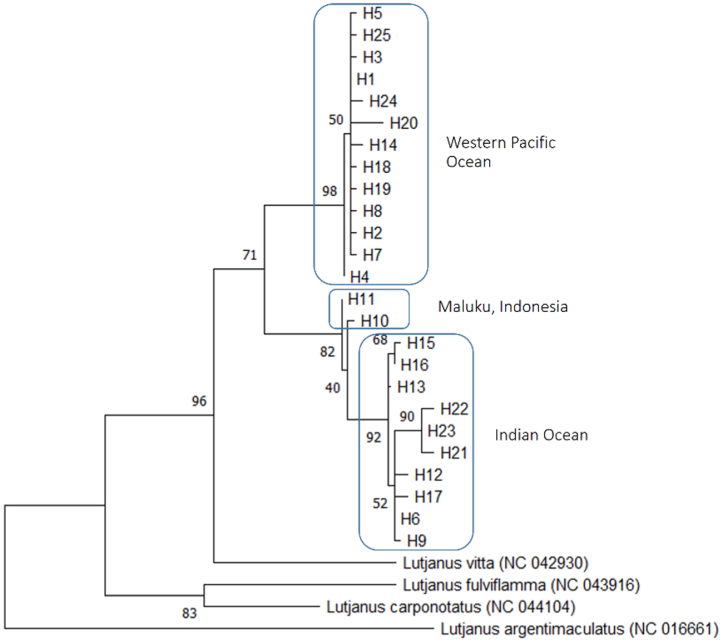
Maximum likelihood (ML) tree using the 25 haplotypes of *Lutjanus lutjanus*. For outgroups, *Lutjanus vitta, Lutjanus fulviflamma, Lutjanus carponotatus, and Lutjanus argentimaculatus* were used, with their GenBank accession numbers shown in parentheses. Bootstrap percentages are shown at the node.

Haplotype analysis found 25 haplotypes (H1–H25 in Table 2 and Fig. 5). Two haplotypes, H1 and H6 were dominant with 25 and 28 samples, respectively, whereas other haplotypes only had 1 or 2 samples only. By looking at the haplotype network (Fig. 5) and ML tree (Fig. 6), the samples from the western Pacific Ocean: Brunei Darussalam, China, Indonesia (Bali, East Java, and Lombok), Japan, eastern Peninsular Malaysia (Kelantan), Philippines, Taiwan and Vietnam, formed one genetic population with a bootstrap value of 98%. The samples from the Indian Ocean also formed another genetic population with a bootstrap value of 92%, which were Bangladesh, India, Indonesia (Aceh), Iran, Mozambique, Myanmar, Pakistan, Saudi Arabia, Sri Lanka and UAE. Four samples from Peninsular Malaysia were also included in this Indian Ocean population but no information is available to specify if these samples were obtained either from the western coast of Peninsular Malaysia (Indian Ocean) or the eastern coast of Peninsular Malaysia (western Pacific Ocean). Interestingly, two samples (H10 and H11) from Maluku, Indonesia, formed a different genetic population.

**Table 2:** List of haplotypes for *L. lutjanus.* *No information is available to specify if the sample was from the eastern or western coast of Peninsular Malaysia.

Haplotype	No. of samples	Localities (sample ID or GenBank accession number)
H1	25	Brunei Darussalam (LV8, LV9, LV11, LX21, LX22), China (EF607552, EF607553, KY371699, FJ237809-FJ237812), Indonesia: East Java (KT781907), Indonesia: Lombok (GU674421, HQ564347, JN311952, JN311953), Japan (LC089003), Taiwan (KU943910, KU943911, KU943882, KU943890, KU943891), Vietnam (MK777451, MK777452)
H2	1	Brunei Darussalam (LX19)
H3	1	Brunei Darussalam (LX20)
H4	2	Brunei Darussalam (LX23), China (KY371697)
H5	1	Brunei Darussalam (LX24)
H6	28	Bangladesh (MH311266, MH311267, MK027396), Indonesia: Aceh (MN257545), Iran (HQ149878, HQ149880), Mozambique (JF493836-JF493841), Pakistan (MN511948-MN511952), Peninsular Malaysia (JN208429)*, Saudi Arabia (KU499715, KX139512, MH331797), United Arab Emirates (UAE; MT076697-MT076701), Myanmar (MH235665, MH235666)
H7	2	China (EF607551, EF607554)
H8	2	China (KY371698), Indonesia: Bali (JN311963)
H9	2	India (KJ920132, KM079315)
H10	1	Indonesia: Maluku (MN870004)
H11	1	Indonesia: Maluku (MN870571)
H12	1	Iran (HQ149879)
H13	2	Pakistan (MN511947), Peninsular Malaysia (JN208431)*
H14	1	Eastern Peninsular Malaysia: Kelantan (MG002621)
H15	1	Peninsular Malaysia (JN208432)*
H16	1	Peninsular Malaysia (JN208430)*
H17	1	Sri Lanka (MT774156)
H18	1	Taiwan (KU943909)
H19	1	Taiwan (KU943908)
H20	1	Taiwan (KU892887)
H21	1	Bangladesh (MK340654)
H22	1	Bangladesh (MK340653)
H23	1	Bangladesh (MK340652)
H24	1	Indonesia: Lombok (GU674418)
H25	1	Philippines (KJ202172)

The nucleotide diversity (π = 0.0020) for the western Pacific population (40 samples; 18 polymorphic sites) of *L. lutjanus* was slightly lower compared to the nucleotide diversity (π = 0.0027) of the Indian Ocean population (39 samples; 15 polymorphic sites). On the other hand, the haplotype diversity was higher in the western Pacific Ocean population (13 haplotypes; diversity = 0.61) compared to the Indian Ocean population (10 haplotypes; diversity = 0.49). The genetic diversity for the Maluku population was not determined due to the small sample size (2 samples). However, the mean genetic distance between the western Pacific and the Indian Ocean was 0.058, indicating that they were highly divergent. Compared with the Maluku population, the western Pacific Ocean population showed a higher between-group mean genetic distance (0.044) than the Indian Ocean population (0.016). AMOVA showed that the between-group variation was higher (95.76%) than the within-group variation (4.24%) for the three genetic populations. If the Maluku population was excluded, AMOVA similarly showed that the between-group variation was higher (95.98%) than the within-group variation (4.02%). The fixation indexes showed that the western Pacific Ocean population was significantly different compared to the Indian Ocean population (F_ST_ = 0.96, p < 0.05). On the other hand, the Maluku population was more different compared to the western Pacific population (F_ST_ = 0.95, p < 0.05) than to the Indian Ocean population (F_ST_ = 0.83, p < 0.05).

## 4 Discussion

The present study is the first to record *L. xanthopinnis* in the waters of Brunei Darussalam and the east coast of Peninsular Malaysia. These two sites are located in the southern South China Sea (Fig. 1), and previous studies on the ichthyofauna of this region have not recorded *L. xanthopinnis*, probably because the species was only recognized as distinct from *L. madras* and other *Lutjanus* species in 2015. *Lutjanus xanthopinnis* and *L. madras* together with other lutjanids occur sympatrically, and these fishes can be differentiated through several morphological features such as colour and scale pattern but their meristic numbers are overlapping among these lutjanids [20]. Therefore, fish species identification often requires proper expertise and extensive experiences to make valid authentication of the specimen, and sometimes identifying specimen at different developmental stages can also lead to confusion and misidentification. Furthermore, additional non-phenotypic information such as habitat and time (season) of collection would be needed for proper species identification, especially when there are low levels of morphological differentiation among species. Indeed, we misidentified 15 specimens from Brunei Darussalam based on their morphological characteristics. These specimens were instead confirmed as *L. lutjanus* using DNA barcoding. Therefore, it is important to conduct species identification using DNA barcoding to validate snappers accurately including the yellow-lined snapper complex.

The study found that the specimens from the eastern Peninsular Malaysia had lower genetic diversity than Brunei Darussalam. This could probably be due to factors such as habitat differences or higher fishing pressure. Haplotype analysis of *L. xanthopinis* showed that some geographically distant localities shared the same haplotypes (Table 1; Fig. 3). The results suggest that spatial genetic structure is absent, and hence, *L. xanthopinnis* might form a panmictic population. Life history and oceanographic characteristics are known to influence the population structure of marine species [21,22], however these factors' relative role in shaping phylogeographic patterns remains undetermined. Ocean currents and the life history of *L. xathopinnis* may be responsible for connecting the species habitats within its distribution range.

The present study also analysed *L. lutjnaus* and found three genetic populations. Interestingly, these populations are linked to their geographical locations, i.e., the western Pacific Ocean, the Indian Ocean and Maluku in Indonesia. A previous study reported that the *L. lutjanus* specimens from the eastern and western coastlines of Peninsular Malaysia were genetically distinct. One group could potentially be considered a new species of *L. lutjanus* [13]. The present study confirmed that the *L. lutjanus* specimens from the western Pacific Ocean and Indian Ocean were highly divergent. In addition, the present study also found that the *L. lutjanus* specimens from Maluku in Indonesia might also be another distinct genetic population. This unique population structure could be brought about by limited migration between the geographical locations. Alternatively, this could reflect the historical population isolation caused by the lowering and rising of sea levels during the late Pleistocene, which is known to shape the biogeography in Southeast Asia [23]. Thus, further study should be conducted by analyzing more samples, especially from Maluku and surrounding sites. Nuclear and other mitochondrial DNA markers should also be analysed to validate the present findings.

Although the current conservation status of the IUCN Red List is Data Deficient for *L. xanthopinnis*, some other snappers have already been categorized as Near Threatened or Vulnerable [8]. This is not surprising considering that snappers including *L. xanthopinnis* are among the most important fish resources and would be exploited for human consumption. The present study could contribute to further research on stock assessment and conservation measures in the future.

## Author contributions

Conceptualization, T.A.; methodology, T.A. and H.T.; validation, T.A. and H.T.; formal analysis, H.T., N.A. and J.J.; investigation, N.A., J.J., S.A. and N.D.Z.; resources, T.A., J.J., S.A., T.N.A,M.J. and A.H.; writing—original draft preparation, T.A. and H.T.; writing—review and editing, T.A and H.T.; supervision, T.A. and H.T.; project administration, T.A.; funding acquisition, T.A. All authors have read and agreed to the published version of the manuscript.

## Declaration of competing interest

The authors declare that they have no known competing financial interests or personal relationships that could have appeared to influence the work reported in this paper.
